# Irreversible electroporation in a case of pancreatic leiomyosarcoma: a novel weapon versus a rare malignancy?

**DOI:** 10.1186/s12957-018-1553-9

**Published:** 2019-01-05

**Authors:** Alexandros Papalampros, Michail G. Vailas, Ioanna Deladetsima, Demetrios Moris, Maria Sotiropoulou, Athanasios Syllaios, Athanasios Petrou, Evangelos Felekouras

**Affiliations:** 1 1st Surgical Department, Athens University School of Medicine,“Laiko” General Hospital, Agiou Thoma 17, 11527 Athens, Greece; 2 Pathology Department, Athens University School of Medicine,“Laiko” General Hospital, Agiou Thoma 17, 11527 Athens, Greece; 3 Department of Surgery, Duke University Medical Center|DUMC, Durham, USA; 4 3rd Surgical Department, Evangelismos General Hospital, Ypsilantou 47, 10676 Athens, Greece; 5 Nicosia Department of Surgery/Div. HPB, 93 Agiou Nikolaou Street, Engomi, 2408 Nicosia, Cyprus

**Keywords:** Irreversible electroporation, IRE, Pancreatic cancer, Pancreatic leiomyosarcoma

## Abstract

**Background:**

Primary pancreatic leiomyosarcoma is an extremely rare entity that needs high clinical suspicion in order to diagnose it at an early stage. Clinical characteristics, diagnosis, and management still remain challenging and controversial, especially in advanced stages, when tumor invades adjacent vessels and organs or gives distant metastases.

**Case presentation:**

Herein, we describe a case of a 57-year-old woman suffering from advanced pancreatic leiomyosarcoma with thrombosis of the superior mesenteric vein, as well as liver lesions which were suspicious for metastasis. Multidisciplinary team decided for upfront chemotherapy to assess tumor response. Follow-up imaging after the completion of chemotherapy led tumor board to decide for subsequent surgical exploration. The patient underwent exploratory laparotomy and irreversible electroporation ablation of the pancreatic tumor. Postoperative course was uneventful, and she was discharged 10 days later with a plan to receive adjuvant therapy. To the best of our knowledge, this is the first case of pancreatic leiomyosarcoma ever reported, treated with this novel technique of irreversible electroporation that could be an alternative and feasible way for the management of these rare malignancies.

**Conclusions:**

In conclusion, primary pancreatic leiomyosarcoma is a rare and highly malignant tumor associated with poor prognosis. Nowadays, R0 surgical resection remains the cornerstone treatment, combined with adjuvant and/or neoadjuvant chemotherapy prior to resection. In the advanced setting, when major vessel invasion and distant metastases occur, chemotherapy along with irreversible electroporation ablation could be a helpful and possibly effective modality for the management of this highly aggressive tumor.

## Background

Pancreatic leiomyosarcoma is an extremely rare malignant entity with only few reported cases in the medical literature. Therefore, the current evidence on diagnosis and management of these rare tumors is based mostly on single case reports and small case series. Pancreatic leiomyosarcomas represent 0.1% of all malignant pancreatic tumors. They seem to be more prevalent in the fifth decade of life, with nonspecific symptoms at their initial presentation [1]. The tumor is usually aggressive in nature, and 25% of patients suffer from distant metastases at the time of diagnosis [2]. Median survival is reported to be around 48 months with a 5-year associated survival of about 43.9% [3]. A complete resection is considered the sole and gold standard treatment for this type of malignancy [1]. However, in cases of advanced and/or metastatic pancreatic leiomyosarcoma, an established consensus for the management of these tumors does not exist, whereas efficacy of adjuvant and neo-adjuvant treatment with chemotherapy and/or radiation remains questionable.

We describe a case of a 57-year-old woman who presented in outpatient clinic suffering from advanced pancreatic leiomyosarcoma, causing thrombosis of the superior mesenteric vein (SMV) and portal vein, along with multiple liver lesions, which were suspicious for malignancy. Multidisciplinary team decided for upfront chemotherapy in order to assess tumor response. Follow-up imaging after the completion of chemotherapy led tumor board to decide for subsequent surgical exploration. Following that, the patient underwent exploratory laparotomy and irreversible electroporation (IRE) ablation of the pancreatic tumor as well as local excision and microwave ablation of liver lesions which were suspicious for metastasis. To the best of our insight, this is a unique case of pancreatic leiomyosarcoma treated with IRE, a novel and innovative weapon for the treatment of several malignancies at advances stages.

## Case presentation

A previously healthy 57-year-old woman, with no significant past medical history, presented to the surgical department of our hospital for definite management of a primary pancreatic leiomyosarcoma, after being treated with adjuvant chemotherapy.

**Fig. 1:**
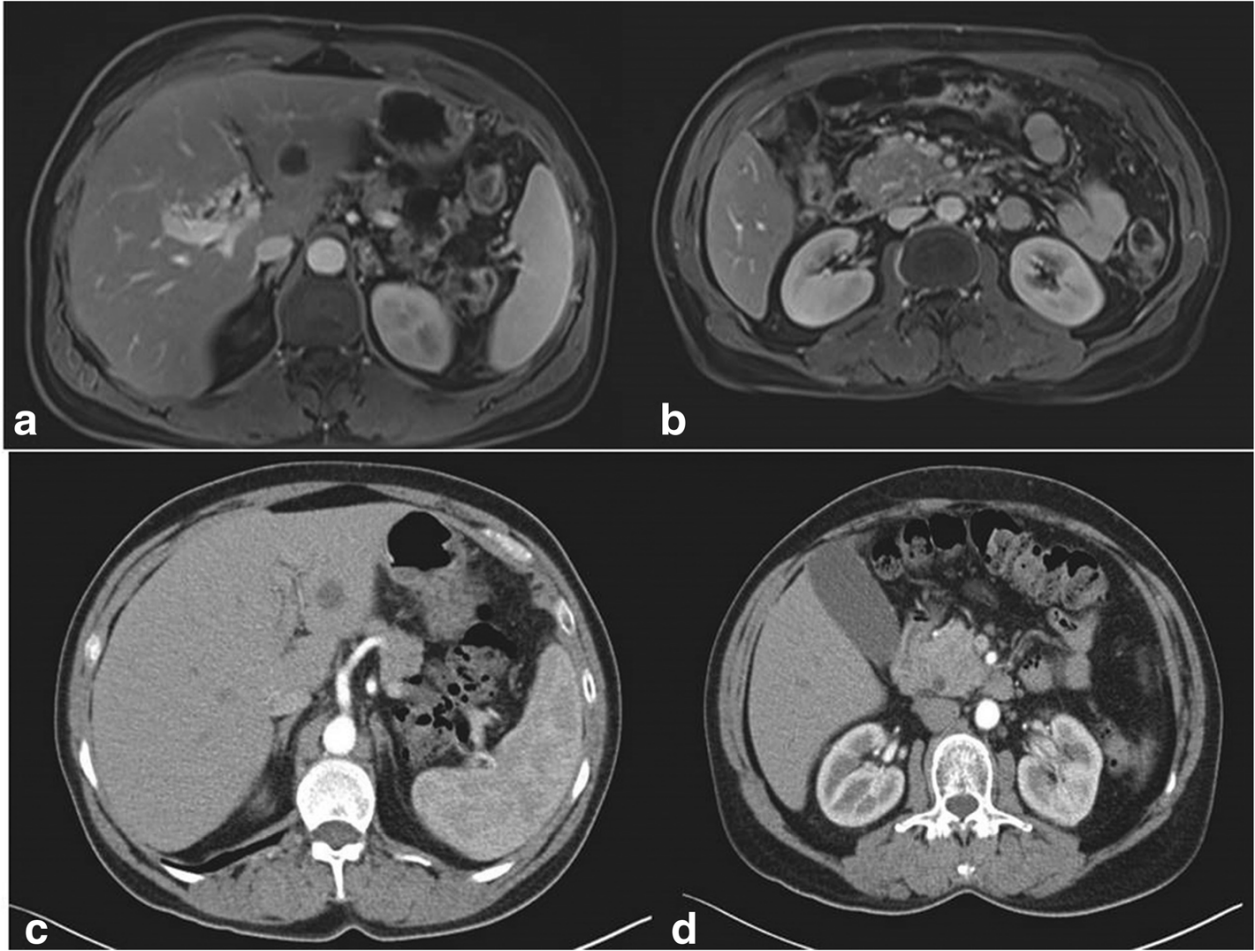
**a** MRI showing suspicious lesion in segment III of the liver prior to chemotherapy. **b** MRI showing pancreatic mass along with thrombosis of SMV prior to chemotherapy. **c** CT showing downsizing and minimal response of the segment III liver lesion after chemotherapy. **d** CT showing downsizing and minimal response of pancreatic mass after chemotherapy

One year before her last admission, she was initially admitted to our emergency department due to abdominal pain, fatigue, and weight loss. She was totally healthy prior to these symptoms. She then underwent magnetic resonance imaging (MRI) that was indicative of a pancreatic head lesion along with possible metastatic liver lesions, superior mesenteric vein occlusion, and portal vein infiltration (Fig. 1a, b). The decision was to undergo an endoscopic ultrasound (EUS) biopsy in order to determine the exact nature of the lesion. EUS report was indicative of pancreatic leiomyosarcoma.

Multidisciplinary team’s decision was to use gemcitabine- and docetaxel-based chemotherapy as up-front treatment to assess tumor response. Follow-up CT scan and magnetic resonance imaging (MRI) after the completion of chemotherapy regimen showed downsizing of the pancreatic mass, as well as downsizing of suspicious for malignancy segment III liver lesion (Fig. 1c, d).

**Fig. 2:**
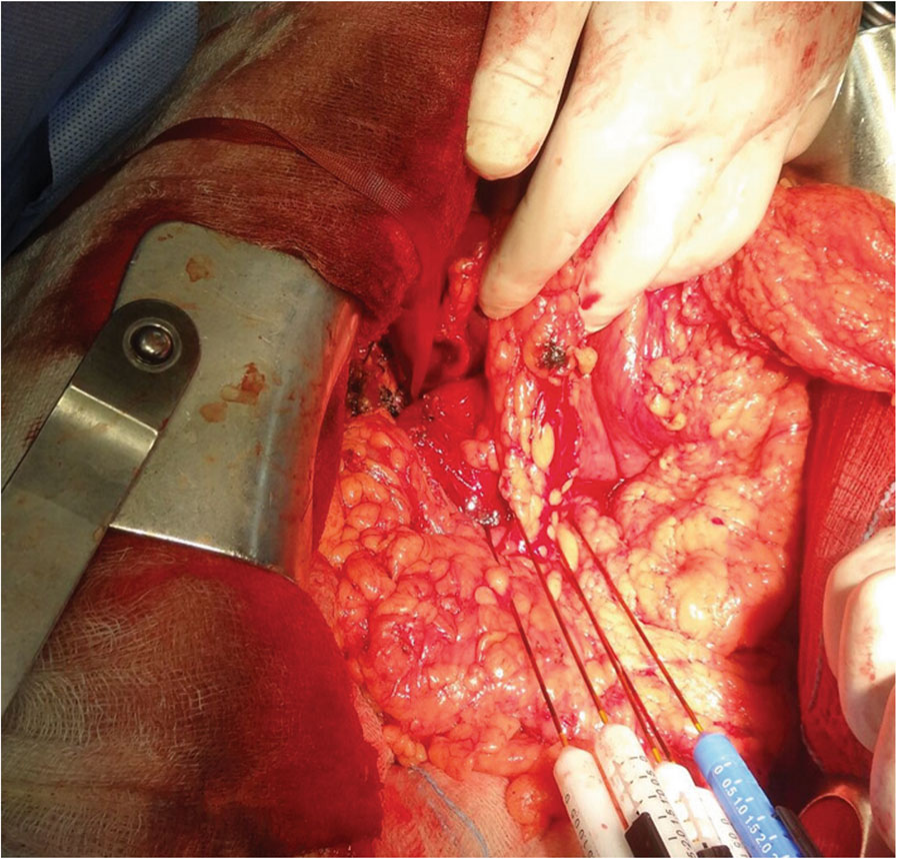
Application of IRE needles in the lesion of the head of the pancreas

Based on the response to chemotherapy, tumor characteristics, and physical status of the patient, multidisciplinary team’s decision was to proceed to surgical exploration. Due to local expansion of the pancreatic tumor, its relation with the superior mesenteric and portal vein, and the underlying SMV thrombosis, excision of the pancreatic tumor was not feasible. Intraoperatively, a small piece of tumor was excised in order to be sent for histopathology. Surgeon’s decision was to ablate the tumor with irreversible electroporation (Fig. 2). Metastatic liver lesions were identified with the use of intraoperative ultrasound. Segment III liver lesion was resected, while smaller lesions of the right lobe were ablated using microwave ablation.

**Fig. 3:**
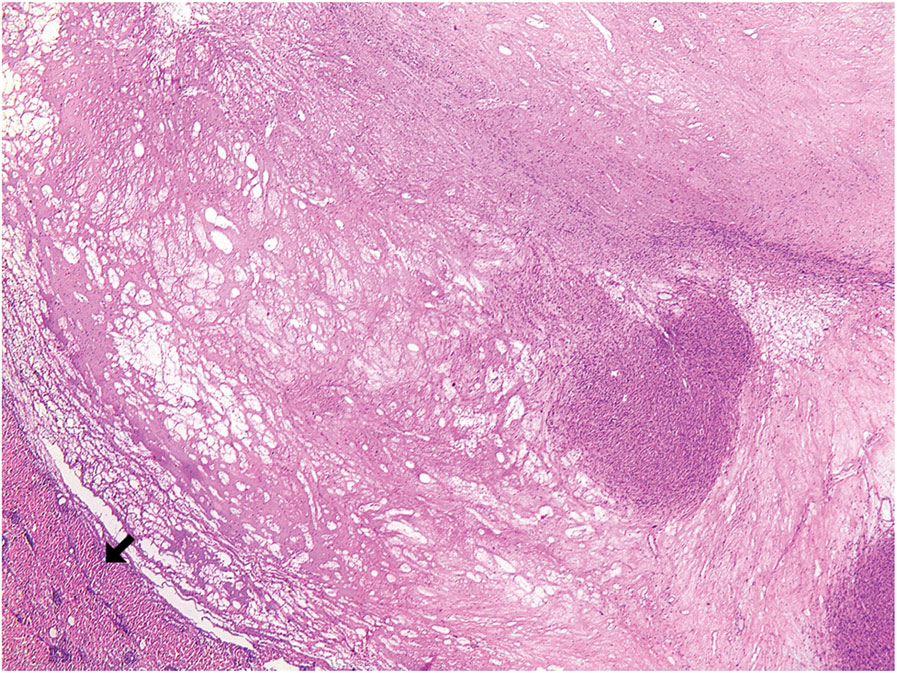
Extensive regression changes with nodular sarcoma remnants. Liver tissue is recognizable at the periphery (arrow), (H-E × 20)

**Fig. 4:**
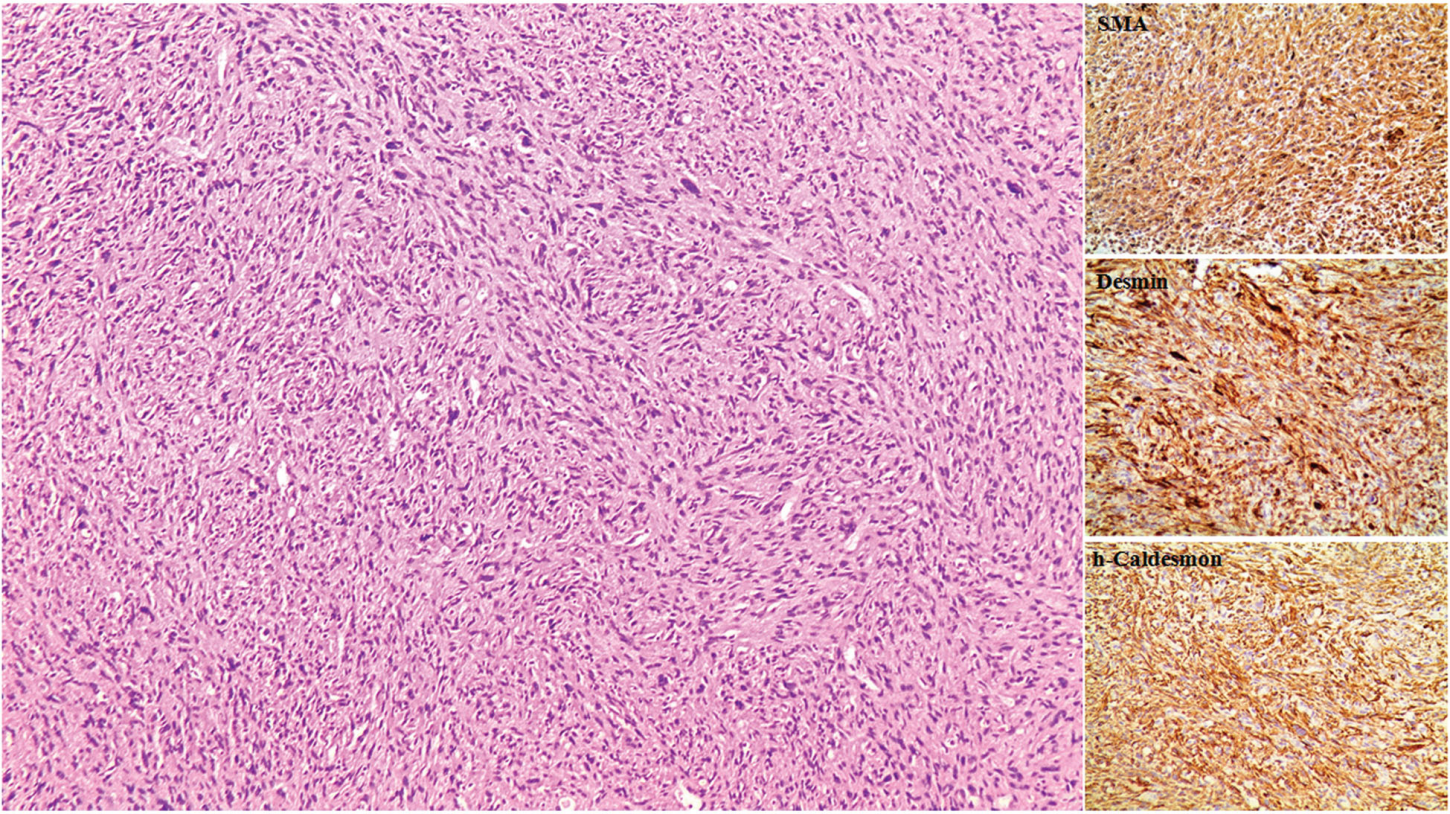
Sarcomatous tissue of high cellularity with fascicular pattern. Multinucleated giant cells can occasionally be seen (H-E × 100). Diffuse cytoplasmic immune reactivity for SMA, desmin and h-Caldesmon, and chromagen DAB (× 200)

**Fig. 5:**
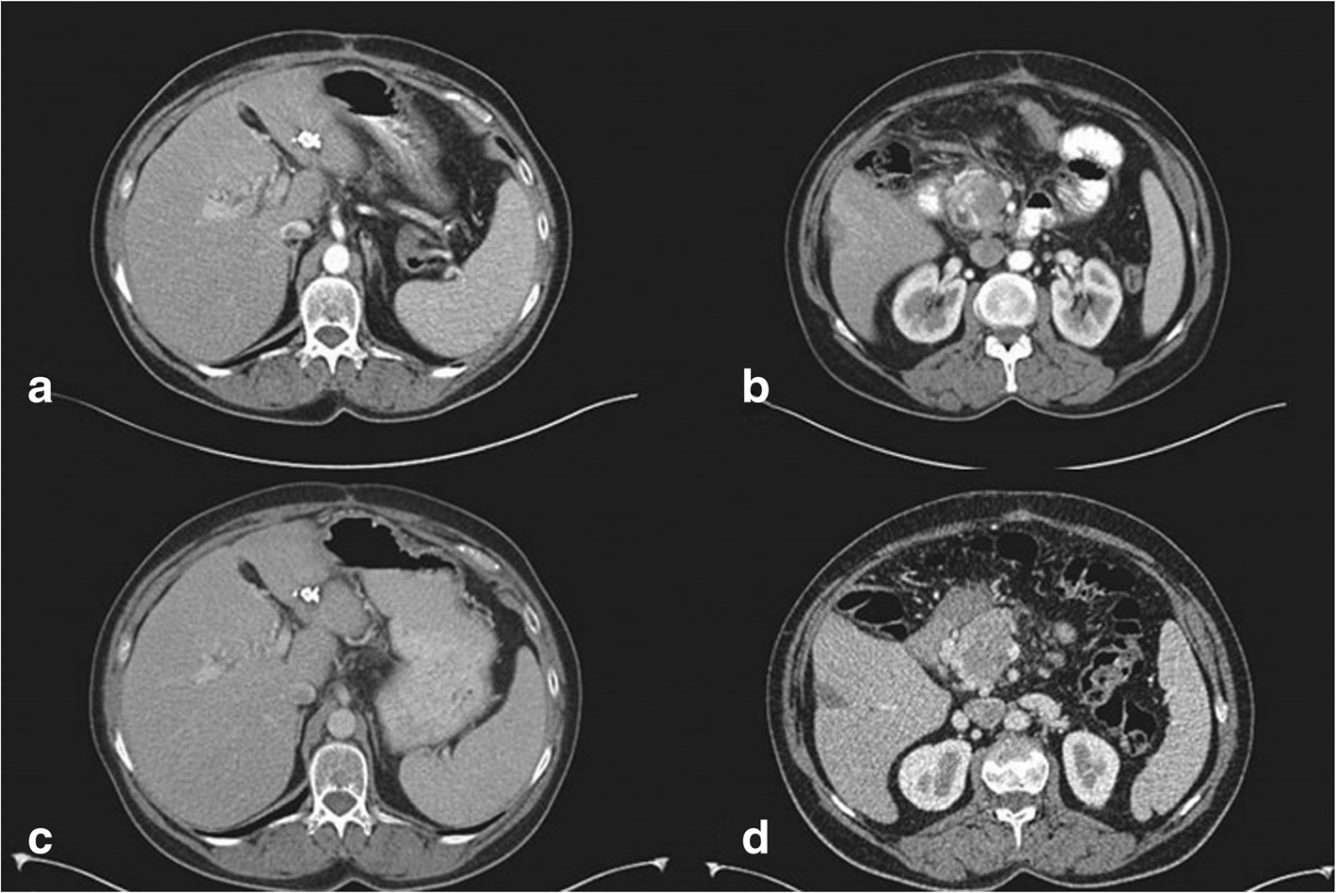
Follow-up CT scan. **a** Site of segment III resection. No new liver lesions detected 6 months after the operation. **b** Pancreatic head lesion after IRE with no obvious changes or progression of disease 6 months after the operation. **c** Twelve months after the operation, the liver remains free of new lesions. **d** Twelve months after the operation, the pancreatic mass shows no progression or enlargement

The patient had an uneventful postoperative recovery and complete resolution of her symptoms. Histopathological examination of pancreatic lesion as well as segment III liver lesion revealed sarcomatous tissue of high cellularity with fascicular pattern, increased mitotic activity, and diffuse cytoplasmic immune reactivity for SMA, desmin and h-Caldesmon, and chromagen DAB (Figs. 3 and 4). Surprisingly enough, pathological report of a smaller liver lesion was indicative of angiomyolipoma staining positive for HMB45 and Melan-A. The lesion was a benign hamartomatous, circumscribed but unencapsulated hepatic mass composed mainly by mature lipocytes and limited mesenchymal component (smooth muscle cells), showing no marked atypia and thick-walled vasculature. Myoid component was positive for ΗΜΒ-45 and Melan-A. Based on the histopathological report, tumor board decided that the patient should be treated with adjuvant therapy for leiomyosarcoma after surgery. A regimen with anthracycline and olaratumab was used for 3 months. Follow-up imaging in 6 and 12 months showed no progression of the disease (Fig. 5a–d).

## Discussion

It is well known that pancreatic adenocarcinoma is the most common histologic type of malignancy of the pancreas, representing more than 90–95% of all pancreatic tumors. Pancreatic sarcomas are encountered with a reported incidence of about 0.1% of all malignant pancreatic tumors, with leiomyosarcoma being the most common type reported [1]. Pancreatic leiomyosarcoma, which was first described by Ross in 1951, is an extremely rare tumor with nonspecific clinical and radiological features and also poor prognosis [2].

Pancreatic leiomyosarcoma seems to be more prevalent in female patients in the fifth decade of life with a variable size at the time of their presentation between 1 and 30 cm [2]. There is no predilection regarding the tumor location (body-tail or head of the pancreas) [1]. It is considered to originate either from the wall of small intra-pancreatic vessels or from smooth muscle cells of the pancreatic duct [2]. At the time of diagnosis, 25% of patients suffer from metastatic disease, whereas invasion to adjacent organs or vessels is reported to be around 19%. The most frequent sites of metastases are the liver and lungs. In contrast, lymph node involvement is rare [3].

Patients are symptomatic in 90% of cases. The most common disease presentations are abdominal mass (50%), non-specific abdominal pain (43%), and weight loss (33%). However, none of the symptoms are specific. Rarely, patients present with jaundice, anemia, gastrointestinal bleeding, and vomiting [1]. As far as tumor morphology is concerned, 53% are solid, 16% are cystic, and 31% have a mixed pattern. Small tumors are usually found incidentally, but large tumors with cystic degeneration are highly aggressive presenting with adjacent organ invasion and distant metastases [4]. As the tumor increases its size, cystic changes can be observed that can be confused with pseudocysts of the pancreas. A fact that is of paramount importance is that the pancreas is often invaded by leiomyosarcomas originating from the duodenum, the stomach, and retroperitoneal organs, mimicking pancreatic leiomyosarcoma. Tumors originating from these organs should be ruled out before the definite diagnosis of pancreatic leiomyosarcoma [4, 5].

Primary pancreatic leiomyosarcoma may be diagnosed securely based on histopathologic morphology [1]. Well-formed fascicles of spindle cells with blunt-ended nuclei intersecting at vertical angles, abundant eosinophilic cytoplasm, varying degrees of pleomorphism, and possible increased mitotic activity may be seen [2]. However, these findings are not pathognomonic and differential diagnosis should include other soft tissue tumors. It can be distinguished from other mesenchymal tumors due to the positive staining for smooth muscle actin and desmin [1, 5]. In our case, these markers stained positive, whereas nuclear stains for MDM-2 as well as for HMB45 and Melan-A were negative, characteristic markers when positive for angiomyolipoma. Grading is also an important prognostic factor as well as an indicator for metastatic risk [1]. Serum tumor markers are usually negative [2].

As there are no specific imaging features, imaging studies cannot distinguish primary pancreatic leiomyosarcoma or its metastases from other tumors of the pancreas. Trans-abdominal ultrasonography, computed tomography, MRI, endoscopic ultrasonography, and angiography are all effective in detecting the tumor [4]. In small leiomyosarcomas, ultrasonography shows a regular hypoechoic pattern [3]. Necrotic, hemorrhagic, or cystic changes can be seen in larger leiomyosarcomas, converting the signal to a mixed echoic pattern. CT may reveal a heterogeneous, hypervascular mass with contrast enhancement in arterial and venous phase [6]. Multidetector CT angiography is also an accurate technique for characterizing pancreatic leiomyosarcomas, due to the hypovascular pattern display in the arterial phase and the homogeneous enhancement in the venous phase [7]. MRI characteristics of leiomyosarcomas include isointensity with skeletal muscle on T1-weighted images and hyperintensity on T2-weighted images [8]. Gadolinium enhancement is usually heterogeneous. FDG-PET scan may reveal an area of increased tumor metabolic activity and a central area of low metabolic activity [1]. Nowadays, EUS has gained broad acceptance because it enables a more imaging-directed biopsy of lesions, becoming an indispensable part of the diagnostic and staging tools. Reyes et al. proposed EUS-guided fine needle aspiration and fine needle biopsy for setting the diagnosis of a primary pancreatic leiomyosarcoma and its liver metastases [9].

The standard treatment for localized sarcomas is surgery with free tumor margins [10]. Non-radical resection is an independent adverse prognostic factor [2]. The tumor size, location, and metastases play pivotal role on the decision for surgery. In small pancreatic tumors, limiting the extent of surgery is feasible [1]. Kocakoc et al. proposed that if the lesion is located in the head of the pancreas, then therapeutic approach is pancreatoduodenectomy; if the lesion is located in the pancreatic corpus or tail, then distal pancreatectomy is suitable, whereas if the patient presents with widespread metastases, then surgery is not suitable and palliative chemotherapy may be offered [8]. Local excision of the pancreatic leiomyosarcoma has also been reported [11]. As there is not an established consensus on the treatment of primary pancreatic leiomyosarcomas, there are no clear indications for adjuvant treatment with chemotherapy and/or radiation. Doxorubicin-based chemotherapy is considered the first-line treatment for leiomyosarcomas, not amenable to curative-intent surgery [12]. Six cycles of doxycycline and ifosfamide as well as gemcitabine-based chemotherapy have been tried in the adjuvant setting [1].

In recent years, irreversible electroporation has been studied for locally advanced pancreatic cancer. Ansari et al. [13] mentioned that IRE has a low post-procedural mortality eliminating the thermal damage to the blood vessels, bile ducts, or other nearby structures, unlike radiofrequency ablation (RFA), which is a well-established technique for thermal ablation of solid tumors in many organs. IRE offers better preservation of vessels, nerves, and extracellular matrix, keeping the vital structures intact and resulting in reduced pancreatic tumor growth and increased survival. Most complications are manageable and self-limiting, besides some reports of severe complications, such as portal thrombosis, pancreatic fistula, and pancreatitis [13].

In our case, despite the fact that the patient presented with metastatic disease, she also had satisfactory response to initial chemotherapy and the tumor board decided to perform exploratory laparotomy. Intraoperatively, the decision was to proceed with destruction of the advanced pancreatic leiomyosarcoma with irreversible electroporation, local excision of the metastasis in segment III of the liver, and microwave ablation of smaller right lobe liver lesions. It is the first case of primary pancreatic leiomyosarcoma with liver metastases that was treated with IRE, avoiding major resections and their higher incidence of postoperative morbidity and mortality. Despite the fact that IRE is not a well-established treatment method for the management of multifocal pancreatic leiomyosarcomas, its implementation and efficacy in our case highlight the necessity for new scientific studies to be conducted, which will thoroughly investigate its use as an alternative and effective tool for their treatment.

Median survival of pancreatic leiomyosarcomas is 48 months with overall 1-, 3-, 5-, and 10-year survival rates of 66.6%, 51.2%, 43.9%, and 29.3%, respectively. Age ≥ 55 years, distant metastases, adjacent organs/vessels invasions, and non-radical resection are considered adverse prognostic factors [4]. Another adverse predictor that has been reported is mitotic counts > 10 mitoses per 10 high-powered fields (HPFs) [14]. Makimoto et al. reported six additional resections for recurrences of a pancreatic leiomyosarcoma, suggesting that intraoperative dissemination and hematogenous metastasis may be implicated in the pathophysiology of tumor recurrence. However, the surgical margin is probably the most important factor for local recurrence [15].

It is worth mentioning that besides pancreatic leiomyosarcoma, pathological assessment of a small liver lesion that was resected revealed a synchronous liver angiomyolipoma, which is also a rare mesenchymal tumor. Angiomyolipomas are mostly benign and also a diagnostic challenge for physicians when conventional diagnostic tools are used. These lesions may require biopsy and have a small risk of recurrence (2.4%) and a mortality rate of 0.8% [16]. When diagnosis of hepatic angiomyolipoma is secured without the need of operation, conservative management with annual imaging may be justified, and resection should be performed only if symptoms occur, if there are inconclusive biopsy results, or there is growth in size in follow-up imaging [16]. In our patient, the lesion seemed suspicious for metastasis. Therefore, we proceeded to its resection.

## Conclusion

In conclusion, primary pancreatic leiomyosarcoma is a rare and highly malignant tumor, associated with poor prognosis. Nowadays, R0 surgical resection remains the cornerstone treatment, combined with adjuvant and/or neoadjuvant chemotherapy, prior to resection. Our presented case of a metastatic pancreatic leiomyosarcoma and the fact that our patient is still alive with no progression of the disease 1 year postoperatively may highlight the necessity for more research and scientific studies to be done, using IRE against this type of malignancies, when surgery is not justified. In our patient with advanced stage disease, chemotherapy along with IRE ablation proved to be a helpful and effective modality for its management.

## Abbreviations

EUS: Endoscopic ultrasound
IRE: Irreversible electroporation
RFA: Radiofrequency ablation
SMV: Superior mesenteric vein
